# Do First Generation Immigrant Adolescents Face Higher Rates of Bullying, Violence and Suicidal Behaviours Than Do Third Generation and Native Born?

**DOI:** 10.1007/s10903-014-0108-6

**Published:** 2014-09-24

**Authors:** Kevin Pottie, Govinda Dahal, Katholiki Georgiades, Kamila Premji, Ghayda Hassan

**Affiliations:** 1 Bruyere Research Institute, University of Ottawa, Ottawa, ON Canada; 2 Institute of Population Health, University of Ottawa, Ottawa, ON Canada; 3 Department of Psychiatry and Behavioural Neurosciences, Offord Centre for Child Studies, McMaster University, Hamilton, ON Canada; 4 Department of Family Medicine, University of Ottawa, Ottawa, ON Canada; 5 Department of Psychology, University of Quebec at Montreal, Montreal, QC Canada

**Keywords:** Bullying, Peer aggression, Suicidal ideation, Intergenerational cultural dissonance, Acculturation, Immigrant adolescent health

## Abstract

We conducted a systematic review to examine first generation immigrant adolescents’ likelihood of experiencing bullying, violence, and suicidal behaviours compared to their later-generation and native born counterparts, and to identify factors that may underlie these risks. Eighteen studies met full inclusion criteria. First generation immigrant adolescents experience higher rate of bullying and peer aggression compared to third generation and native counterparts. Refugee status and advanced parental age were associated with increased parent to child aggression among South East Asians. Family cohesion was associated with lower rates of violence. Suicidal ideation was lower across most immigrant adolescents’ ethnicities, with the exception of Turkish and South Asian Surinamese female adolescents in the Netherlands. Bullying and peer aggression of immigrant children and adolescents and potential mitigating factors such as family cohesion warrant research and program attention by policymakers, teachers and parents.

## Introduction

International migration has doubled over the past 30 years [1]. Each year, tens of millions of children and adolescents immigrate to new communities in new countries with or to join their parents [2]. With the risks of facing socioeconomic challenges, [3] as well as straining local resources and engendering anti-migrant sentiments, [2] this scale of migration has implications for the safety and security of immigrant adolescents, with adolescents defined here as aged 10–19 years [4].

One example of risk to safety and security for immigrant adolescents is bullying and peer aggression. These forms of violence have emerged as particular concerns, with studies out of Australia, North America and Europe demonstrating risks to immigrant adolescents [5]. In one American qualitative study, more than 50 % of Asian and South East Asian immigrant adolescents reported ethnic and racial tension and related peer aggression [6]. In immigration contexts, the parents’ task in transmitting their values of origin becomes both more important and more difficult especially when the host culture’s values are perceived as competing and more attractive to the adolescents [7, 17], with resulting intergenerational cultural dissonance. The losses inherent in migration are further compounded by what Robben [8] calls ‘negative social mirroring,’ which refers to the negative image of migrant groups that the majority may convey [9]. This may have implications for the identity and mental health of immigrant adolescents [10]. Victimized ethnic/racial minority adolescents are at risk for social anxiety and depressive symptoms [11, 12]. In the general population, several studies have shown an association between bullying and suicide-related behaviors, [1, 2, 13–15] and a study found evidence consistent with a causal link, at least for girls [16, 17].

Immigrant adolescents may be particularly vulnerable to safety and security risks when there is conflict between familial and school or societal expectations [18]. In a study of Vietnamese and Cambodian immigrant adolescents in the U.S., such conflict indirectly predicted problem behaviours [18]. McQueen et al. [19] found similar results among Mexican–American adolescents, with family conflict aggravating the effects of acculturation on marijuana use and deviant behavior such as theft and engagement in violent activity.

Despite evidence demonstrating increased risk of immigrant adolescents to bullying, peer aggression, and mental health problems, there is little explicit information comparing the experiences of new immigrant adolescents with their later-generation counterparts [20–22]. Our review seeks to address this gap by examining the likelihood of new immigrant adolescents to experience bullying and peer aggression, other forms of violence (including parent-to-child aggression and sexual abuse), and suicidal behaviours. We also sought to identify potential moderators of these risks and variations across immigrant groups. We define first generation immigrant adolescents as those born in the country of cultural origin and who have immigrated with their parents to a new host country. Second generation immigrant adolescents are those born in the host country to at least one immigrant parent. Third generation immigrants are born in the host country to parents who were born in the host country. Our comparison groups were non-immigrant or third generation immigrant adolescents living in the same community [20–22].

This review aimed to address two research questions: Do first generation immigrant adolescents face a higher likelihood of bullying, aggression, and violence than their third generation or native born counterparts? Do first generation immigrant adolescents face a higher likelihood of suicide and suicidal ideation compared to third generation and native born counterparts?

## Methods

The methodology for this systematic review was based on the six stage framework outlined by the Cochrane Handbook for Systematic Reviews of Interventions [23]: (1) identifying the research question; (2) developing search protocol (3) identifying relevant studies based on search protocol; (4) study selection; (5) charting the data; (6) collecting, summarizing and reporting the results. In addition, we consulted immigrant parents from Latin American, Nepalese, Somali ethnic communities in Canada through Café Scientifique meetings to ensure relevancy of our selected outcomes. Concept mapping and synonym listing was prepared based on Population (immigrant and refugee adolescents, and family), Exposure (experiences of acculturation, cultural discordance (differences related to language, cultural beliefs, and religious beliefs), and/or intergenerational cultural dissonance (conflict between parent and adolescent over cultural values), Comparison (versus non-immigrant and later generation immigrants and native born adolescents), and Outcomes (based on the literature identified in our Introduction: bullying, peer aggression, physical or sexual violence, and suicide or suicidal ideation).

### Search and Selection

A sensitive search of electronic bibliographic databases was performed to retrieve all articles combining the concepts of cultural discordance, immigrant and refugee children and adolescents, and safety and security. The search strategy was devised on Ovid Medline and then adapted for other databases. Without limiting by language, we identified studies by searching the Cochrane Library, Medline, Global Health, Health and Safety Science Abstracts, HealthStar, Scopus, PsychInfo, and Social Science Abstracts. In addition, we conducted a sensitive search using the term “intergenerational cultural dissonance.” In all cases, the databases were searched from inception to Oct 31, 2011, and an updating Medline search for new studies with comparison groups was conducted on July 1, 2013. All references were imported into an EndNote Library and tagged with the name of the database. Duplicates were removed within EndNote, leaving the final total of results at 2,274 (2,270 from the electronic databases and 4 from other sources).

Two reviewers independently screened titles and abstracts of all 2,274 studies, applying inclusion and exclusion criteria and identifying relevant articles. Inclusion criteria included observational designs, a sample of greater than 100 participants, and reporting on our outcome variables and our target populations: immigrant adolescents aged 10–19 years and with a comparison population to third generations or native born adolescents. Discrepancies were resolved by consensus. However, if consensus could not be made between two reviewers, a third party expert was involved for tiebreaking. This occurred in only three instances.

Papers which met criteria were then subject to a full text review. To ensure scientific quality, we appraised the studies using the Newcastle Tool for Observation Studies [24] (see Table 3 in Appendix).

### Certainty of Effect Assessment

Two reviewers independently appraised and extracted details of the selected articles using standardised abstraction forms, and resolved discrepancies by consensus. A final quality assessment of all selected studies was conducted using the GRADE (Grading of Recommendations Assessment, Development and Evaluation) approach [25]. We report the quality rating for outcomes in the results. We used a narrative synthesis method to integrate related findings into descriptive summaries [23]. The reporting of the search and selection results followed the PRISMA principles (Preferred Reporting Items for Systematic Reviews and Meta-Analyses) [26].

## Results

### Data Collection and Study Characteristics

**Fig. 1:**
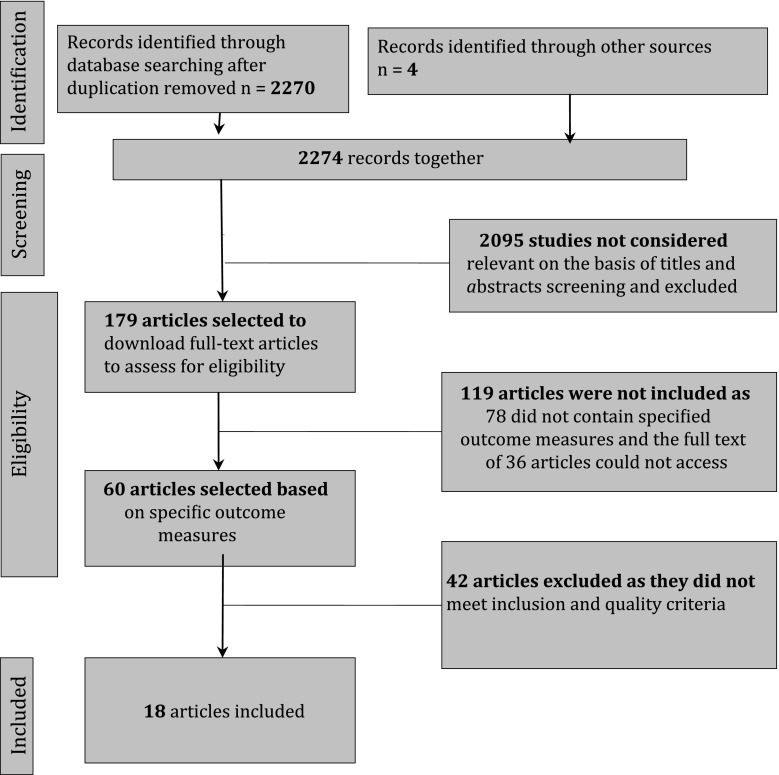
PRISMA flow diagram

A flow diagram (Fig. 1) shows the identification, screening, eligibility assessment, and inclusion of studies in the systematic review. Of the initial 2,274 studies, 179 met our criteria for full appraisal. After repeated appraisals, 18 studies met full inclusion criteria.

Of the 18 studies, a majority were carried out in the USA (14 studies), followed by the Netherlands (2 studies), France (1 study), and Israel (1 study). All studies examined immigrant adolescents where there was a difference in primary language and culture from the local non-immigrant community. There were a total of 41,476 participants across the 18 studies and approximately 65 % of participants were females (Table 1).

**Table 1:** Characteristics of included studies

Study	Study design	Country	Participants	Outcomes measured
Altschul and Lee [35]	Longitudinal	USA	Total n = 845 Hispanic mothers of preschool aged children Foreign-born = 328 Native US born = 517 (Age of mothers not specified)	Maternal physical aggression directed toward young children
Cho and Haslam [40]	Cross sectional	USA	Total n = 227 adolescents Korean immigrants to US = 62 Korean students living in Korea = 47 Non-Korean-American students living in the US = 31 Mean age = 16.6 years (SD = 1.1) Girls = 129 and Boys = 98	Relationship between acculturative stress, social support and suicide- related phenomena among adolescent immigrants in the US
Decker et al. [37]	Cross-sectional	USA	Total n considered for analysis = 5,919 high school girls, White = 76 %, Hispanic 10 %; Black 7 %; Asian 3 %; Others 2 %; Age = 14 years or younger (12 %), 15 years (26 %), 16 years (26 %), 17 years (23 %), 18 years and above (14 %); Immigrants = 13 %	Immigrant status and sexual assault
Fuligni [33]	Both cross-sectional and longitudinal	USA	Total n = 998 students; Longitudinal = 353 and Cross sectional = 645; Sample from: Mexican = 168 (52 % girls) Chinese = 148 (56 % girls) Filipino = 403 (51 % girls) European = 279 (51 % girls); Mean age range = 12.1–15.2 years)	Adolescent’s beliefs, expectations, and relationships with parents on the basis of parental authority and individual autonomy.
Juang and Alvarez [34]	Cross-sectional	USA	Total n = 309 Chinese American students (63 % girls) US born = 66 % Foreign born-29 % Grew-up with both parents = 89 % Age range = 13–17 years	Perceived discrimination with family conflict and family cohesion
Kim et al. [43]	Cross sectional	USA	Total n = 444 Chinese American families Girls = 226; Boys = 218 Age range = 12–15 years	Testing that generational dissonance may indicate a family context that places children at increased risk for adverse outcomes such as acculturation, parenting, adolescent depressive symptoms, discrimination, SES and mother’s and father’s length of stay in the U.S.
Lau et al. [27]	Cross-sectional	USA	Total n = 1,293 Asian American parents of 18 years and older who had at least one child (sex distribution of sample population not reported)	Minor assault (pushed, grabbled, or shoved; threw something at; slapped, hit or spanked) Severe assault (kicked, bit, or hit with fist; beat up; choked; burned or scalded)
Le and Stockdale [28]	Cross Sectional	USA	Total n = 329 Chinese and Southeast Asian youth; Cambodian = 120; Chinese = 64; Lao/Mien = 67; and Vietnamese = 86); Age range = 10–18 years old; Gender—fairly equally distributed.	Acculturative dissonance, ethnic identity, peer delinquency, and serious violence
Molnar et al. [32]	Longitudinal (cohort)	USA	Total n = 8,872 residents from African American, Non-Hispanic White, and Hispanic Age: 3–15 years Gender = boys 50 %, Girls 50 %	Correlation between neighbourhoods and the incidence of parent-to-child physical aggression
Peguero [31]	Longitudinal	USA	Total n = 1,457 Public Latino students; Boys 48.5 % and Girls 51.5 %; Age range- not specifically mentioned; First generation immigrant 27.9 % Second generation immigrant 40.8 % and Third generation immigrant 31.3 %	Role of immigrant status and English proficiency for Latino students’ experiences with school violence: (1) property victimization (2) Violent (3) Fear (feel unsafe), and (4) Formal disciplinary school sections (suspended/put on propagation, and transfer to school from disciplinary reasons
Pena et al. [38]	Longitudinal	USA	Total n = 3,135 Latino adolescents of 16 years where 50 % were females. Of total sample, 25.3 %, comes from 1st generation 40.9 % from 2nd and 33.8 % from 3rd generation Mexican-American 49.9 % Puerto Rican 17.8 %, Cuban-American 15.8 %, Other Hispanics 16.5 %	Relation between suicide attempts and immigrant generation status, also measured predicted risk factors associated with elevated suicide behaviours, namely substance use, problematic alcohol use, and depressive symptoms.
Ponizovsky and Ritsner [41]	Cross sectional	Israel	Total n = 406 Jewish immigrant adolescent to Israel and native-born Jewish Age = 11–18 years Male sample 51.3 %; Mean age of respondents 14.5 (SD 2,1 years)	Examine suicidal ideation and suicide attempts, behaviour problems, psychological distress, social support, and adjustment difficulties in a sample of adolescent
Peguero [30]	Longitudinal (Multivariate regression analysis)	USA	Total n = 8,383 students Latino 1,628, Asian American 1,129, and white American 5,626; 1st generation 28 %, 2nd generation 41 % and 3rd plus generation 31 %; Female 52 %, but age is not specifically mentioned.	Pattern of victimization in schools, in part based on immigrant generation
Spencer and Le [29]	Cross Sectional	USA	Total n = 329 Cambodian = 120 of 15 years Chinese = 64 of 14 years Lao/Mien = 67 of 15 years Vietnamese = 86 of 14 years In all samples, the ratio of males and females fairly equally distributed	Peer delinquency, parental engagement, serious violence and family/partner violence
Van Bergen et al. [39]	Longitudinal	Netherlands	Total n = 4,527 of young females Dutch = 3,090; Moroccan = 557; Turkish = 614; Suriname = 266 Age = 14–16 years	The prevalence of non-fatal suicidal behaviour of the sampled population and examines risk factors in non-western female immigrant adolescents compared to majority female adolescents
Van Bergen et al. [65]	Longitudinal	Netherlands	Total n = 249 Turkish adolescents Age = 12–18 years Female sample 53 %; Male 47 %	The prevalence of suicidal ideation and the vulnerability across several ethnic minorities versus ethnic majority adolescents Also examine whether ethnic minority adolescents are at risk for suicidal ideation because of a family background of migrants, social-economic position and certain family factors – influence psychological constellations.
Van Leeuwen [56]	Cross sectional	France	Total n = 292 students French high school girls = 122; French high school boys = 170 Age = 15–21 years	Role of acculturation in suicidal ideation among second generation immigrant adolescents in France
Ying and Han [42]	Longitudinal	USA	Total n (wave 1) = 5,262 adolescents Total n (wave 2) = 4,288 adolescents Vietnamese 48.4 % Laotian 26.3 % Cambodian 16.5 % Hmong 8.8 % Males 50.4 % and Females 49.6 %	Intergenerational conflict and depressive symptomatology

#### Bullying, Peer Aggression and Violence

Eight U.S.-based studies (four cross sectional and four prospective cohort) examined immigrant status and its associations with victimization, dating violence, school violence, parent-to-adolescent physical aggression, peer discrimination and harassment, violence, and sexual violence.

First generation immigrant adolescents from non-English speaking countries reported more episodes of bullying and violence at school than did non-immigrant adolescents. This was noted among Cambodian, Chinese, Lao/Mien and Vietnamese [27–29], Latino [30–32], and Filipino [33] populations. First generation Latino students were more likely to report being a victim of violence than third generation (English speaking) Latino students (*p* < 0.001) [31]. Discrimination was associated with loneliness, anxiety and somatization, but among Chinese populations, immigrant family cohesion buffered the negative effects of discrimination [34].

First generation Latino American immigrant adolescents were more likely to feel unsafe at school than later generation Latinos [31]. There was a strong positive association between students feeling safe in their learning environments and educational success among Latino and Asian first generation immigrant adolescents; the third generation adolescents of immigrant origin usually reported feeling safer than their first generation counterparts but less so than their White counterparts [30].

First generation Latin immigrant mothers demonstrated lower rates of parent-to-adolescent aggression than did native born Latin American mothers. Immigrant status reduced risk, while maternal alcohol use, parenting stress, and child aggression emerged as the strongest risk factors for maternal physical aggression [35]. Chicago neighbourhoods with greater concentrations of immigrants, independent of the characteristics of immigrant families, had a lower rate of parent to adolescent aggression [32]. Parents’ refugee status, however, was associated with increased family violence in another study of South Asian adolescents [29]. Older parental age was associated with severe parent to adolescent aggression in one Asian American immigrant study (OR 1.08; *p* < 0.05) [27]. In a Korean American study, risk for parent-adolescent physical aggression from immigrant mothers was found to increase when the immigrant mothers themselves experienced high acculturation conflict and discrimination in host societies [36].

In the U.S., first generation immigrant girls who were black adolescents or sexually active Hispanics experienced higher rates of sexual assault among age and ethnic/racial groups [37]. Acculturation did not seem to impact risk of sexual victimization [37].

#### Suicide and Suicidal Ideation

Eight studies (four cross sectional studies and four prospective cohort studies) examined suicide and suicidal ideation among immigrant adolescents. These studies measured acculturation stress, depression or depressive symptoms, distress, social and attention problem, academic performance, suicidal ideation, and suicide attempts among immigrant adolescents.

In a U.S. national survey of more than 20,000 Latino American adolescents/adolescents, suicide attempts were less likely among first generation Latino Americans, with second generation Latinos having a higher RR at 2.87 (95 % CI 1.34–6.14) and third generations having the highest likelihood with a RR 3.57 [38]. These findings of lower rates of suicidal ideation and fewer suicide attempts among earlier generations were also found in studies examining Korean American [39, 40], Latin American [41], Russian Israeli [38], and Moroccan Dutch [39] immigrant adolescents. Notable exceptions were South Asian-Surinamese and Turkish female immigrant adolescents in the Netherlands, who experienced a higher rate of suicide attempts (19.2 and 14.8 % respectively) than non-immigrant Dutch adolescents (9 %) and Moroccan immigrant adolescents (6.2 %) [39].

Mitigating and aggravating factors for suicidal ideation and suicide were also identified. In the U.S., perceived child–parent discrepancy in preference for American ways significantly predicted intergenerational cultural dissonance (β = 0.27; *p* = 0.05), and this conflict in acculturation during early adolescence predicted increased depressive symptomatology in late adolescence [42]. In a Korean immigrant study from the U.S., acculturation stress was associated with increased psychological symptoms (*p* < 0.047) and suicidal ideation (*p* < 0.01) [32]. Among these adolescents, living in an intact family was associated with markedly better outcomes with respect to suicidal ideation than when a family was geographically separated [40]. In a Chinese American immigrant study, a high discrepancy in father to adolescent acculturation levels was associated with significantly more adolescent depressive symptoms, as was unsupportive father to adolescent relationships. The sample included slightly more daughters than sons. Adolescent reports of parental warmth are associated with less depressive symptoms (*p* < 0.001) [43] (Table 2).

**Table 2:** Summary of Findings- Bullying, peer aggression, sexual violence and suicide among immigrant children and adolescents

Outcome	Studies and Participants	Summary of Findings	GRADE Estimate of certainty of evidence
Suicide attempts/ideation	8 observational studies Korean, Latin American, Jewish, Russian, Suriname, Moroccan and Turkish, Chinese	First generation immigrant adolescents had lower suicide attempt rates than non-immigrant adolescents and third generation immigrant adolescents (RR 2.87; 95 %, CI 1.34–6.14) Immigrant adolescents who are not living together with their biological parents reported higher levels of life stress and suicidal thoughts than their counterparts who were living with parents	Low
Bullying, Peer Aggression Sexual violence and other violence	10 observational studies Cambodian, Chinese Vietnamese, Latin American, Laos, Asian American, Filipino	First generation and non-native English speaking immigrants were more likely to report of being victim of violence at school than native English speaker counterparts. (*p* < 0.001) Parent to child aggression was lower in first generation immigrant families in US compared to White and Black American families. Refugee status and advanced parental age were associated with increased parent to child aggression among South East Asians	Low

## Discussion

To our knowledge, this study is the first international systematic review examining the effect of generational status on violence and suicidal behaviours experienced by immigrant children and adolescents. Previous reviews examining such outcomes have focused more on aspects of criminality and delinquency [44].

We found that across many immigrant groups, bullying and peer aggression were consistently significantly higher for non-official language speaking first generation immigrant adolescents compared to third generation and native born adolescents. This suggests that risks related to violence are greater when an immigrant adolescent speaks a language other than the primary language of the host country. This highlights a distinct sensitive period during migration [45, 46] that demands ongoing research to disaggregate data on socioeconomic status, gender, and ethnicity. Aggravating factors may include high academic standing [47], while alleviating factors may include safe schools [48], ethnic diversity within schools [49, 50], and family cohesion [34]. These findings seem to reflect previously proposed frameworks within which intergenerational cultural dissonance contributes to the violence experienced by immigrant adolescents [27, 34]. Immigrant parents are faced with the challenge of ensuring the continuity and transmission of their cultural heritage, while simultaneously promoting their adolescents’ integration into the host culture. However, when both cultural environments, family cultural of origin and host culture promote conflicting values, the result may be increased intergenerational cultural dissonance, family conflict and increased risk for violence [17, 51, 52]. Scholars’ have encouraged immigrant parents’ to transmit their cultural values of origin, as a protective factor [53]. Conversely, undermining immigrant parents’ transmission of values may contribute to a rise of family conflict and adolescent difficulties.

Studies in the general population have associated bullying with health related symptoms [54], and likewise, in immigrant populations, peer aggression and ethnic bullying have been repeatedly associated with negative repercussions for mental health [16]. However, efforts to study immigrant adolescent health outcomes internationally are just beginning [55]. Although immigrant adolescents are very heterogeneous both within and across countries, the process of facing discrimination and adapting to new cultural, language and other norms includes shared commonalities.

Immigrant adolescents appeared to benefit from cohesive families, and despite evidence for acculturation stress and family intergenerational cultural dissonance, suicidal behaviour rates remained low among immigrant adolescents, and lower among first generation immigrant adolescents than their later generation counterparts. Immigrant adolescents who are not living together with their biological parents experience a higher level of life stress and suicidal thoughts than their counterparts living with intact families [32]. Repeated use of drugs other than marijuana and alcohol may be another potential mediator of generation status on suicide attempts [30], as might low socioeconomic status and poor academic performance [56]. Among South Asian-Suriname and Turkish adolescent girls in Holland, it appeared that uncommonly high rates of violence within the families may have played a significant role in a higher risk of suicidal behaviours among this population [39]. Finally, despite the risks, the likelihood of suicide was low and researchers have argued that immigrant children and adolescents may escape local risk due to cultural values and supportive family environments [57, 58].

Our review uncovered one study related to sexual assault experienced by immigrant girls, with first generation black adolescent girls and sexually Hispanic girls being at increased risk in the U.S. [32]. Though this was the only study in our review documenting increased risk of sexual violence among specific subpopulations of immigrant adolescents, sexual violence and victimisation against women and girls is internationally acknowledged as a health, social, political, and human rights concern, particularly within precarious refugee or immigration contexts [59–61]. The reasons for increased vulnerability are multiple and cross all components of their eco-system. For example, cultural tensions within families may constitute such vulnerability and increase the risk of victimisation for immigrant girls for several reasons including lack of appropriate sexual education on strategies to detect danger and self-protect for first generation immigrant girls. Risk may also increase in contexts where sexual harassment is silenced due to stigma and the resultant feelings of shame, self-condemnation, and distrust that constitute major barriers to disclosure [62]. In addition, the higher victimisation of black girls may be understood within a framework of racism.

Our findings reinforce previous studies demonstrating that most immigrant adolescents endure psychosocial stress when integrating into their new country. However, while data on risk would suggest poor mental health outcomes, our review found that this was largely not the case. This supports a large body of literature documenting an immigrant mental health paradox or advantage, whereby despite exposure to psychosocial and economic adversity, immigrant youth in Canada, the U.S., the U.K., and Australia generally have better mental health, compared to non-immigrant youth [20–22, 63].

## Limitations

There are important limitations to this review. First, data came from observational studies which cannot infer causality. The certainty of this evidence was low based on GRADE appraisals. Second, we considered published research on adolescents and parents, but did not include research on teachers, religious and community group leaders and administrative officers who provide services for adolescents. Perspectives from these groups may be worth examining. Third, there is a need for more consistency in terminology related to immigrant status in order to track adolescents over time to study the impact of immigration on psychological outcomes [64].

Finally, our studies came from multiple countries and considered multiple immigration nationalities and there was also heterogeneity of outcomes. Although we could identify a few outlying ethnic- or country-specific effects, more are likely to be present. In some of the studies, immigrant subgroups (refugees and immigrants) and cultural groups (Asian, Latin American) were grouped together, and the data was not always disaggregated to look at potential equity issues during analysis. This heterogeneity and aggregation of outcomes reduced the certainty of our conclusions and negated any pooling of the data.

## Conclusions

The findings of this review identified that adolescents from first generation non-native language speaking backgrounds (Chinese, Cambodian, Vietnamese, and Latin American) were more likely to experience victimization from bullying and peer aggression at school than later generation immigrants. Despite these challenges, most immigrant adolescents living with their biological parents had lower rates of suicidal ideation and suicide attempts compared to their non-immigrant and native-born or second- and third generation counterparts. Studies showed that in most cases, a supportive, cohesive (all members living together) family is associated with both less violence and less suicidal ideation.

Ongoing research is needed examining the challenges experienced by immigrant adolescents and their families, as well as the mediating and mitigating factors associated with these challenges. Research must continue to include ethnically diverse samples and different immigration statuses and other variables, and subgroup analyses are needed in order to better understand differences among and between groups. Further studies may also benefit from examining the vulnerability factors associated with increased risk of being bullied, and developing and assessing the effectiveness of programs aimed at prevention or intervention.
